# Factors associated with frequent use of emergency-department services in a geriatric population: a systematic review

**DOI:** 10.1186/s12877-019-1197-9

**Published:** 2019-07-05

**Authors:** Isabelle Dufour, Maud-Christine Chouinard, Nicole Dubuc, Jérémie Beaudin, Sarah Lafontaine, Catherine Hudon

**Affiliations:** 1 Faculté de médecine et des sciences de la santé, Université de Sherbrooke, 2500 Boul. de l’Université, Sherbrooke, Québec J1K 2R1 Canada; 2 Département des sciences de la santé, Université du Québec à Chicoutimi, 555 Boul. de l’Université, Chicoutimi, Québec G7H 2B1 Canada

**Keywords:** Review (MeSH), Emergency service, Hospital (MeSH), Frequent use, Aged (MeSH)

## Abstract

**Background:**

Frequent geriatric users of emergency departments (EDs) constitute a small group of individuals accounting for a disproportionately high number of ED visits. In addition to overcrowding, this situation might result in a less appropriate response to health needs and negative health impacts. Geriatric patients turn to EDs for a variety of reasons. A better understanding of the variables associated with frequent ED use will help implement interventions best suited for their needs**.**

**Objective:**

This review aimed at identifying variables associated with frequent ED use by older adults.

**Methods:**

For this systematic review, we searched Medline, CINAHL, Healthstar, and PsyINFO (before June 2018). Articles written in English or French meeting these criteria were included: targeting a population aged 65 years or older, reporting on frequent ED use, using an observational study design and multivariate regression analysis. The search was supplemented by manually examining the reference lists of relevant studies. Independent reviewers identified articles for inclusion, extracted data, and assessed quality with the *JBI Critical Appraisal Checklist for Studies Reporting Prevalence*. A narrative synthesis was done to combine the study results. A sensitivity analysis was performed to evaluate the effect of removing the studies not meeting the quality criteria.

**Results:**

Out of 5096 references, 8 met our inclusion criteria. A high number of past hospital and ED admissions, living in a rural area adjacent to an urban center, low income, a high number of prescribed drugs, and a history of heart disease were associated with frequent ED use among older adults. In addition, having a principal-care physician and living in a remote rural area were associated with fewer ED visits. Some variables recognized in the literature as influencing ED use among older adults received scant consideration, such as comorbidity, dementia, and considerations related to primary-care and community settings.

**Conclusion:**

Further studies should bridge the gap in understanding and give a more global portrait by adding important personal variables such as dementia, organizational variables such as use of community and primary care, and contextual variables such as social and economic frailty.

**Electronic supplementary material:**

The online version of this article (10.1186/s12877-019-1197-9) contains supplementary material, which is available to authorized users.

## Background

Population aging significantly impacts the use of healthcare services, notably emergency departments (EDs). A certain proportion of seniors are considered frequent ED users [1], which are a minority of patients using a major proportion of ED services over a given period [2]. Frequent users aged 65 years or older represent as little as 6% of all ED patients but can account for up to 28% of its accesses [3, 4]. Frequent ED use increases the risk of adverse effects such as hospitalizations, functional decline, complications related to treatment and procedures, and suboptimal follow-up [5, 6]. Older adults visiting EDs are more prone to experience misalignment between their medical needs and their use of health care. Unmet medical needs can lead older adults to use these services instead of more adequate ones [7].

Currently, there is a lack of consensus regarding the definition of frequent ED users. Definitions generally range from patients with two to 12 and more ED visits per year [3, 8]. However, the most common definition for ED frequent use is four or more visits within a year period [2, 9]. Many reasons have been raised to explain seniors’ frequent ED use, including avoidable visits for nonurgent problems [10, 11]. Nevertheless, seniors present more vulnerability factors, as well as more chronic conditions and complex medical needs [5]. The ED is at the core of healthcare for acutely ill seniors, who tend to present a higher priority score than younger adults [12].

Frequent geriatric ED users are far from being a homogeneous population. Identifying their numerous individual characteristics could improve our understanding of how their medical and social needs, as well as their healthcare use, might be best managed and assisted. Even if many studies, including scoping and systematic reviews, have examined frequent ED use among the adult population, few seem to focus on older adults [1, 13]. McCusker et al. (2003) aimed at identifying determinants of ED use among older people. Their main results report that need factors figure among the main determinants of older adults’ ED use. These factors include perceived health status, specific diagnoses (heart disease, diabetes, psychiatric disorders), and composite measures of comorbidity [14]. No recent review has specifically examined frequent ED use among older adults. This systematic review aimed at identifying variables associated with frequent ED use by older adults.

## Methods

A systematic review was conducted in accordance with the Joanna Briggs Institute (JBI) guidelines [15]. Reporting was made in accordance with the *Preferred Reporting Items for Systematic Reviews and Meta-Analyses (PRISMA) Checklist*, presented in Additional file 1 [16].

### Eligibility criteria

English- and French-language studies were included if they: (1) targeted a population aged 65 years or older; (2) reported frequent ED use; (3) used an observational study design–which included prospective and retrospective cohort studies, case-control studies, cross-sectional studies, case series, and case reports; and (4) used multivariate regression analysis to reveal variables associated with frequent ED use.

### Information sources and search strategy

A science librarian helped with a bibliographic search of studies published before June 2018 in the following online databases: Medical Literature Analysis and Retrieval System (MEDLINE), Cumulative Index to Nursing and Allied Health Literature (CINAHL), HealthStar, and Psychological Information (PsyInfo).

The search strategy used medical-subject-heading (MeSH) terms and keywords related to a geriatric population (aged, aged 80 and over, older, elder, geriatrics, senior, Limitation: 65 + years), to frequent users (frequent users, frequent attend*, frequent consult*, frequent use*, high utiliz*, high consult*, high attend*, high use*, repeat use*, repeat, recidivist*, revolving door, misuse, hyperuse, super use*), and to ED use (emergenc*). The terms were also matched with Boolean operators (AND, OR) within the database. The search strategy can be found in the Additional file 2. To enhance the search strategy and examine additional sources, we included hand searching through reference lists in pertinent studies.

### Study selection and data extraction

First, all screened literature was imported into Endnote (Thomson Reuters, Philadelphia, PA, USA) to facilitate organization and removal of duplicates. After duplicates were removed, two reviewers independently screened titles and abstracts (ID and SL), then full texts (ID, CH or MCC) for eligibility. One reviewer (ID) examined reference lists in pertinent studies to identify additional relevant studies. Two reviewers (ID and JB) used the *JBI Data Extraction Form for Prevalence and Incidence Studies* [15] to extract data from eligible studies, to which was added the following information: definition of frequent ED users, sample size, inclusion/exclusion criteria, and variables associated with frequent ED use. During the whole process, uncertainties were resolved through team discussions and consensus.

### Quality appraisal and data synthesis

Two reviewers (ID and JB) independently assessed the risk of bias using the *JBI Critical Appraisal Checklist for Studies Reporting Prevalence Data* [15]. Any discordance was resolved by a third party. The *Prevalence Critical Appraisal Tool*’s purpose is “to determine the extent to which a study has addressed the possibility of bias in its design, conduct and analysis” (Munn et al., 2017; p.2). The tool includes nine questions; the overall appraisal results were *Include*, *Exclude*, or *Seek further info*. The decision to include a study was based on authors judgment and consensus. The tool considered information such as sample frame and size, coverage, and appropriateness of the conducted analysis. A narrative synthesis was used to combine the results of the studies [17]. A sensitivity analysis was performed to evaluate the impact on the study’s conclusions of removing the studies that failed to meet the quality criteria of the *Prevalence Critical Appraisal Tool* [18, 19].

## Results

### Study selection

#### Search results

**Fig. 1:**
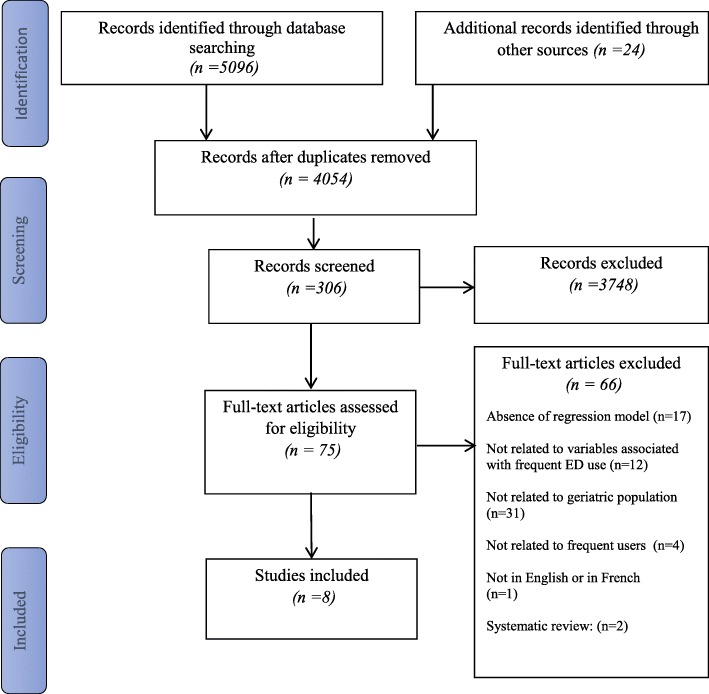
Prisma flow diagram of search results

The search yielded 5096 articles. After removal of duplicates, 4054 articles remained; their titles and abstracts were examined. Of these articles, 75 were screened by full text and eight met the eligibility criteria. Figure 1 provides the PRISMA flow diagram of the search results.

### Study characteristics

#### Included studies

Table 1 gives the characteristics of the included articles. The publication year of the eight articles ranged from 1987 to 2016. Two studies took place in the United States, three in Canada, two in Italy, and one in Ireland. Studies were presented as an observational cohort study [20], retrospective and prospective cohort studies [21–24], and cross-sectional studies [25]. The design was not clearly mentioned in two of the studies [26, 27] but was identified as observational by three of the authors (ID, CH, and MCC).

**Table 1:** Characteristics of included studies

References	Authors	Year of Publication	Country	Study Design	Population	Sample Size	Data source	Definition of Frequent ED use
[27]	Franchi et al.	2016	Italy	*NM Observational*	Community-dwelling subjects aged 65 years or older managed by general practitioners	1,949,020	Administrative database	4 or more ED visits within a year
[23]	Lishner et al.	2000	USA	Retrospective study	Washington State residents aged 65 years or older who were Medicare beneficiaries in 1994 and did not belong to a capitated health plan	354,782	Administrative database	Patients with 5 or more ED outpatient visits
[20]	McCusker et al.	2000	Canada	Cohort study	Patients aged 65 years or older who visited the EDs in one of Montreal’s four hospitals.	1122	Administrative database	1) 3 or more episodes of ED care during a 6-month period
[21]	McCusker et al.	1997	Canada	Cohort study	Patients aged 75 or older who visited an ED in a Montreal hospital and those whose first visit was during the index period, and who were aged 65 or older	Assessment sample113 Retrospective survey 4466	Questionnaire Administrative database	Repeat visits within 90 days
[26]	Naughton et al.	2010	Ireland	*NM Observational*	Elderly patients from EDs at Dublin’s two teaching hospitals	306	Questionnaire	One or more ED visits within a 6-month period
[25]	Parboosing et al.	1987	Canada	Cross-sectional	Elderly patients of the ED of a Calgary hospital	75	Questionnaire	Number of ED visits within a 6-month period
[24]	Rosenblatt et al.	2000	USA	Retrospective study	Washington State residents aged 65 years or older who were Medicare beneficiaries and did not belong to a capitated healthcare plan	354,782	Administrative database	5 or more outpatient ED visits within a 12-month period
[22]	Sona et al.	2012	Italy	Prospective study	Patients aged 65 years or older from an ED in a Turin hospital	1632	Questionnaire	2 or more ED visits within a 12-month period

### Quality assessment

Overall, the quality of the included studies was adequate. Only one was rated as having inadequate quality because of the convenience sample and the lack of statistical power, limiting the conduction of meaningful analysis [21]. The sensitivity analysis, however, indicated that its removal did not alter the results.

### Study description

#### Population, sample size, and data sources

Most of the studies included a population aged 65 years or older, except one [21], which recruited a population aged 75 years or older. All the studies focused on the population’s general characteristics. Sample size varied from 75 to 1,949,020 people. Data sources for the measures of associated variables also differed: four studies used administrative databases [20, 23, 24, 27], three used questionnaires [22, 25, 26], and one a combination of both methods [21]. Two studies used the same administrative database [23, 24].

#### Definition of frequent users

The outcome variables had to target a measure of frequent ED use. There was no consensus on the definition of frequent users: seven different definitions were given in the eight articles. Studies considered the number of ED visits within periods ranging from 3 months to a year [21–27].

#### Associated variables

Multinomial logistic regression models with a 0.05 significance level were used in all the studies. Table 2 provides the variables associated with frequent ED visits among elderly users.

**Table 2:** Variables associated with frequent ED visits

Variables	Details	Number of Studies Using this Variable	Number of Studies in Which the Variable Was Significant	References for Significant Studies
Healthcare-services use				
Hospital admission	Past hospital admission	8	6	[20, 23–27]
Emergency department	Past ED visit	4	2	[20, 27]
Level of general practitioner’s workload	Low N of managed patients	1	1	[27]
	Low or moderate % of elderly managed	1	1	[27]
Other	Attitude towards health-service use	1	1	[25]
	More than one source of healthcare	1	1	[25]
Demographic				
Age	Older age	8	3	[23, 24, 27]
Sex/gender	Being male	8	2	[21, 27]
Location	Rural residence adjacent to an urban center	2	2	[23, 24]
	ED within 10 km from place of residence	1	1	[27]
Socioeconomic				
Social support	Lack of support	4	2	[20, 26]
Housing status	Living alone	5	1	[21]
Family status	Married Widowed	3	1	[20]
Income	Medicaid coverage	3	2	[23, 24]
Health status				
Prescribed drugs	Number of drugs prescribed	3	2	[22, 27]
Mental illness	Depression	3	1	[20]
Alcohol consumption	Less than daily	1	1	[20]
Physical diseases	Heart disease	2	2	[20, 22]
	History of diabetes	1	1	[20]
	Respiratory disorder	1	1	[20]
	Pulmonary neoplasm	2	1	[22]
Number of conditions	Number of functional problems	2	1	[21]
	Number of active diseases	2	1	[22]
Measure of comorbidity	Case mix	2	2	[23, 24]
ED-discharge diagnosis	Digestive	3	1	[20]
Questionnaire score	Physical ability Per unit increase	1	1	[26]
	Anxiety Per unit increase	1	1	[26]
	Condition of partial or complete dependence (Activities of daily living)	2	1	[22]
	Score on the Identification of Seniors at Risk screening tool (ISAR)	1	1	[20]

#### Healthcare-services use

A higher number of past hospital admissions was a significant variable in six out of the eight studies that included this variable [20, 23–27]. A higher number of past ED visits was another variable significantly associated with frequent ED use by seniors. Indeed, this variable was significant in two of the four studies that included it [20, 27]. In contrast, having a principal care physician (generalist or specialist) was considered a protective factor and was significant in the two studies that included this variable [23, 24].

#### Demographic variables

Older age was significantly associated with frequent ED visits in only three studies, while all the articles considered this variable [23, 24, 27]. In addition, all the studies included sex, with being male a significant variable in only two [21, 27]. Three studies considered location and suggested that living within 10 km of an ED [27] or in a rural residence adjacent to an urban center [23, 24] was associated with frequent ED use. In contrast, two studies considered that older adults living in remote rural residences were less likely to be identified as frequent ED users [23, 24].

#### Socioeconomic variable

Variables related to lack of social support were included in four studies and significant in two of them [20, 26]. Income-related variables (Medicaid beneficiary) were considered in four studies and significant in two of them [23, 24].

#### Variables related to health status

A greater number of prescribed drugs was included in three studies and significant in two of them [22, 27]. Moreover, studies reported that some physical and mental disorders were associated with higher rates of ED use by older adults [20, 22, 27]. More importantly, having a heart disease [20, 22] was significant in the two studies that took this variable into account.

## Discussion

This systematic review identified factors most often associated with frequent ED use by older adults: a high number of previous hospital and ED admissions, living in a rural area adjacent to an urban center, low income (Medicaid beneficiary), high number of prescribed drugs, and history of heart disease. Variables associated with a lack of social support yielded mitigated results, while others—such as comorbidity, dementia, and primary-care-related variables—returned few results or were not accounted for.

First, the results emphasize the impact of past ED visits and hospitalizations. Directly related to seniors’ health, they were among the most cited risk factors [14, 28]. As an example, Andersen’s *Behavioral Model of Health Services Use* is commonly used to account for the use of healthcare services among the older population. It indicates that need factors—including past use of healthcare services—are the most important variables influencing healthcare use [14, 29]. Notions related to the length of stay in hospital EDs, while known as having deleterious effects, were not, however, considered in the included studies [30]. As an example, older adults with frailty or severe health conditions are more prone to longer hospital stays. This puts them at greater risk of functional decline, associated with ED returns and rehospitalizations [31].

Our results also indicate residence location as being a demographic variable associated with frequent ED use. Older adults living in rural areas tend to report poorer health status and more healthcare issues than their peers in urban areas [32]. Indeed, people from adjacent rural areas tend to have more ED visits primarily because of the limited availability of care services. In addition, since service availability is generally limited, older adults in remote rural areas use fewer ED services. The availability of healthcare services also varies according to the type of rural area [32].

Being a Medicaid beneficiary came out as an important variable. Nevertheless, it received scant attention in studies looking at ED use by older adults. Income information is not usually provided in databases, while Medicaid insurance status—used as a proxy for socioeconomic status [14, 33]—can only be considered in studies conducted in the United States. Otherwise, income can be considered an indicator of unmet health needs; some authors indicate that these needs should be considered instead. Indeed, seniors with lower incomes or on Medicaid insurance might be more likely to report such unmet needs and to turn to healthcare services such as EDs in fulfilling them [34].

In addition, the risk of using several drugs simultaneously is increased as a result of multiple chronic conditions, which frequently occurs in older adults [35]. Polypharmacy is a proxy for comorbidity severity and can therefore be considered a variable associated with frequent ED use by older adults [36]. Indeed, multimorbidity is associated with medication intake, and the number of medications tends to be proportional to health status [37]. Conversely, having comorbidities and being a major user of health services exposes patients to polypharmacy, among other things [36]. Only two studies adjusted for case mix [23, 24], and none considered a composite measure of comorbidity. Controlling for these would yield a more accurate representation of the population of frequent geriatric ED users. Moreover, morbidity clustering is considered a better predictor of healthcare-services use than a specific disease [38]. In our results, heart disease was the most important specific disease associated with frequent use. Indeed, older adults with such conditions are considered at greater risk of health deterioration. They are then more likely to use EDs and become frequent users of these services [28].

For its part, social support yielded mitigated results. A systematic review by Valtorta et al. (2018) pointed out that there is no clear evidence of a relationship between social support and ED use by older adults. This can be explained by the multidimensional aspects of this concept and the many variables used to measure it (e.g., homelessness, dwelling type, social network, or perceived social network). Social support is related to social functioning, a broader concept covering, among other forms of social participation, social networks, social resources, and social relationships [39]. Pinsonnault et al. (2009) stated that assessing social functioning might be useful in documenting the needs for interventions in older adults [40]. In fact, subsequent studies should then attempt to consider all facets of social functioning to better represent its effects [39].

Additionally, some other important variables received only little or no consideration. First, none of the studies included seniors’ specific living environments. Because of their lower health status, older adults living in private residential-care facilities are more predisposed to ED visits and hospitalization, compared to those living at home or in an independent-living facility [41]. Moreover, variables related to community health services, such as home healthcare, were not considered. If home healthcare can contribute to improving older adults’ health management, this clientele is considered at higher risk of ED visits, related to their higher multimorbidity level [42, 43].

Little consideration was given to variables related to mental health, which were reported as one of the main reasons older adults visit EDs [44]. The mental-disorder category includes various conditions, and some, particularly dementia, would benefit from being explored independently. According to an integrative review by Hunt (2018), individuals with dementia had consistently higher rates of ED visits [45]. Dementia induces vulnerability and implies greater comorbidity, hospitalization episodes, and mortality rates among older adults [35]. Mental-health considerations would therefore be no less important than physical disease in analyzing frequent ED use by older adults.

Variables related to primary-care settings variables were also understudied. Adequate primary-care follow-up can help prevent complications, as well as a certain proportion of ED visits by older adults [46]. As reported in our results, having a primary-care provider plays a role in managing seniors’ health issues and positively affects their ED use [47]. Beyond that, however, care must be accessible and ongoing. For instance, in the United States, states with high rates of ED visits tend to report gaps in access to primary care [48]. Patients with limited continuity of care tend to report more visits to these services, as well as an increased risk of ED visits and hospitalizations [49]. So, unmet health needs may persist even with a higher number of primary-care visits. A study by Horney et al. (2012) points out that older adults who were frequent ED users had more prior visits to primary-care physicians [50]. The number of visits might then be more important than the fact of having a primary-care provider. Therefore, measuring continuity and access to primary care should be considered.

This study has clinical and policy implications. Indeed, pooling the most recent information–even if scarce–on this population is necessary to monitor trends in frequent ED use and to help implement accurate interventions. In fact, case management strategies, additional support systems, and better access to care would have the benefit of improving the health of frequent geriatric users of ED and may contribute to lower their use of these services. As suggested by Pines et al. (2012), creating an effective categorization of frequent users could represent a promising avenue, making it easier to compare and group studies. Categorization would also bring relevant information to existing risk-assessment methods, direct the development of new ones, and raise situations where better care coordination is needed.

### Strengths and limitations

The use of a systematic review method is one of the study’s strength. Despite the small number of articles included, most of them focused on nationally representative samples, thereby improving the scope of the results. In addition, a rigorous search strategy helped acquire the main aspects of the topic. However, the results outlined several definitions of frequent ED users, complicating comparisons among studies. Secondly, variables deemed important in the literature were not included. This is partly due to data source–databases and questionnaires–inducing limitations on the availability of certain variables. Since grey literature was omitted, relevant articles could have been missed.

## Conclusion

This systematic review identified the main factors associated with frequent ED use by older adults: a high number of past hospital and ED admissions, living in a rural area adjacent to an urban center, low income, high number of prescribed drugs, and a history of heart disease. Further studies should bridge the gap in understanding and give a more global portrait by adding important personal variables such as dementia, organizational variables such as use of community and primary care, and contextual variables such as social and economic frailty.

## Additional files

Additional file 1: Prisma Checklist. (DOC 64 kb)

Additional file 2: Search strategy. (DOCX 14 kb)

## Data Availability

All data generated or analyzed during this study are included in this. published article.

## Abbreviations

ED: Emergency department
JBI: Joanna Briggs Institute
PRISMA: Preferred Reporting Items for Systematic Reviews and Meta-Analyses
