# Supplementary material for: Factors associated with frequent use of emergency-department services in a geriatric population: a systematic review
Source: BMC Geriatr. 2019 Jul 5;19:185. doi: 10.1186/s12877-019-1197-9 (PMC6610907; doi:10.1186/s12877-019-1197-9)
Supplement: Supplementary file 2 — Search strategy. (DOCX 14 kb) [file 12877_2019_1197_MOESM2_ESM.docx]

**Additional file 2.** Search strategy.

**Medline**

((Limiters- Aged groups Aged: 65+ years) AND ("Frequent users" OR "Frequent attend*" OR "Frequent consult*" OR "Frequent use*" OR "High utilz*" OR "High consult*" OR "High attend*" OR "High use*" OR "Repeat use*" OR "Frequent flyer*" OR "Heavy use*" OR "Repeat*" OR "Recidivist*" OR "Revolving door" OR "Misuse" OR "Hyperuse" OR "Super use*") AND "Emergen*"))

**CINAHL**

((Limiters- Aged groups Aged: 65+ years) AND ("Frequent users" OR "Frequent attend*" OR "Frequent consult*" OR "Frequent use*" OR "High utilz*" OR "High consult*" OR "High attend*" OR "High use*" OR "Repeat use*" OR "Frequent flyer*" OR "Heavy use*" OR "Repeat*" OR "Recidivist*" OR "Revolving door" OR "Misuse" OR "Hyperuse" OR "Super use*") AND "Emergen*"))

**Health Star**

((Aged AND Ages 80 and over [mesh]) AND ("Frequent users" OR "Frequent attend*" OR "Frequent consult*" OR "Frequent use*" OR "High utilz*" OR "High consult*" OR "High attend*" OR "High use*" OR "Repeat use*" OR "Frequent flyer*" OR "Heavy use*" OR "Repeat*" OR "Recidivist*" OR "Revolving door" OR "Misuse" OR "Hyperuse" OR "Super use*") AND "Emergen*"))

**PsycINFO**

((Aged OR older OR elder OR geriatric OR senior) AND ("Frequent users" OR "Frequent attend*" OR "Frequent consult*" OR "Frequent use*" OR "High utilz*" OR "High consult*" OR "High attend*" OR "High use*" OR "Repeat use*" OR "Frequent flyer*" OR "Heavy use*" OR "Repeat*" OR "Recidivist*" OR "Revolving door" OR "Misuse" OR "Hyperuse" OR "Super use*") AND "Emergen*"))
